# Working draft genome sequence of the mesophilic acetate oxidizing bacterium *Syntrophaceticus schinkii* strain Sp3

**DOI:** 10.1186/s40793-015-0092-z

**Published:** 2015-11-11

**Authors:** Shahid Manzoor, Bettina Müller, Adnan Niazi, Anna Schnürer, Erik Bongcam-Rudloff

**Affiliations:** Department of Animal Breeding and Genetics Science, Swedish University of Agricultural Science, SLU-Global Bioinformatics Centre, Uppsala, SE 750 07 Sweden; Department of Microbiology, Swedish University of Agricultural Sciences, BioCenter, Uppsala, SE 750 07 Sweden; University of the Punjab, Lahore, Pakistan

**Keywords:** Syntrophic acetate oxidizing bacteria, Acetogens, Syntrophy, Methanogens, Hydrogen producer, Methane production

## Abstract

*Syntrophaceticus schinkii* strain Sp3 is a mesophilic syntrophic acetate oxidizing bacterium, belonging to the *Clostridia* class within the phylum *Firmicutes*, originally isolated from a mesophilic methanogenic digester. It has been shown to oxidize acetate in co-cultivation with hydrogenotrophic methanogens forming methane. The draft genome shows a total size of 3,196,921 bp, encoding 3,688 open reading frames, which includes 3,445 predicted protein-encoding genes and 55 RNA genes. Here, we are presenting assembly and annotation features as well as basic genomic properties of the type strain Sp3.

**Electronic supplementary material:**

The online version of this article (doi:10.1186/s40793-015-0092-z) contains supplementary material, which is available to authorized users.

## Introduction

During anaerobic degradation of organic material, acetate is formed as a main fermentation product, which is further converted to methane. Two mechanisms for methane formation from acetate have been described: The first one is carried out by aceticlastic methanogens converting acetate to methane and CO_2_ under low ammonia conditions [1]. The second mechanism, dominating under high ammonia conditions, occurs in two steps, and is performed by acetate-oxidizing bacteria oxidizing acetate to H_2_ (formate) and CO_2_ and a methanogenic partner using the hydrogen (formate) to reduce CO_2_ to methane [2–4]. Most fascinating on this syntrophic relationship is, that the overall reaction operates with a ΔG°´ of -36 kJ x mol^−1^ close to the thermodynamic equilibrium.

The number of isolated and characterized SAOB is restricted most likely due to their considerable differences in substrate utilization abilities and cultivation requirements. To date three mesophilic SAOB, namely *Clostridium ultunense* [5], *Syntrophaceticus schinkii* [6], “*Tepidanaerobacter acetatoxydans**”* [7] and two thermophilic SAOB, namely *Thermacetogenium phaeum* [2] and *Thermotoga lettingae* [8] currently renamed to *Pseudothermotoga lettingae* have been isolated and characterized. Among those, two complete genome sequences of *T. phaeum* [9], *“T. acetatoxydans”* [10] and one draft genome sequence of *C. ultunense* [11] have been published, the later two by this working group. Here, we are presenting the draft genome sequence of the third mesophilic SAOB *Syntrophaceticus schinkii* strain Sp3. To date, strain Sp3 is the only isolated and characterized representative of the species *S. schinkii* and was recovered from an up flow anaerobic filter treating wastewater from a fishmeal factory [6]. This process was characterized by high ammonium concentration (6.4 g l^−1^ NH_4_^+^). *S. schinkii* shows the least narrow substrate spectrum compared to all known SAOB, when growing heterotrophically [6]. The main end product formed is acetate, what allocates the species to the physiological group of acetogens.

Since the recovery of *S. schinkii* we found it at high abundance in all mesophilic large scale and lab scale biogas producing process we have investigated so far. Genome analysis and comparative genomics might help us to understand general features of syntrophy in particular energy conservation and electron transfer mechanisms during syntrophic acetate oxidation.

The present study summarizes genome sequencing, assembly and annotation as well as general genomic properties of the *Syntrophaceticus schinkii* strain Sp3 genome.

## Organism information

### Classification and features

**Fig. 1:**
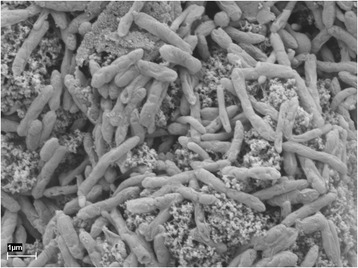
Image. Phase-contrast micrograph of *Syntrophaceticus schinkii* strain Sp3

*Syntrophaceticus schinkii* Sp3 (Fig. 1) is an obligate anaerobic, endospores forming bacterium, whose cells were found to be Gram variable with changing shapes dependent on the growth condition (Table 1, [6]). No flagella have been observed under any condition tested. It can grow up to 0.6 M NH_4_Cl in pure culture between 25 °C and 40 °C. A more detailed physiological description can be found in Westerholm et al. [6]. Minimum Information about the Genome Sequence (MIGS) of *S. schinkii* strain Sp3 is provided in Table 1 and Table S1 (Additional file 1).

**Fig. 2:**
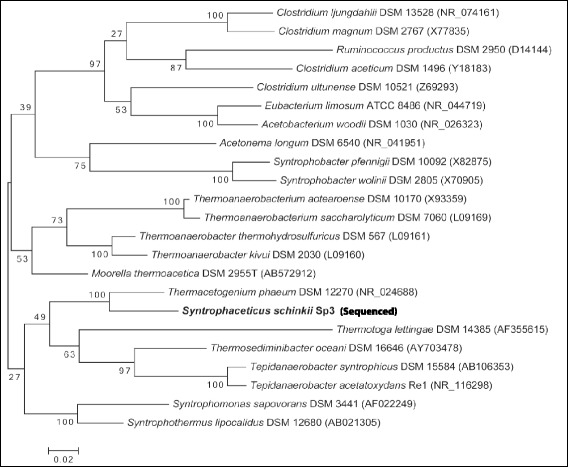
Phylogentic tree. Phylogenetic tree highlighting the relationship of *Syntrophaceticus schinkii* Sp3 relative to known SAOB, acetogens, and other syntrophic operating bacteria. The 16S rRNA-based alignment was carried out using MUSCLE [32] and the phylogenetic tree was inferred from 1,521 aligned characteristics of the 16S rRNA gene sequence using the maximum-likelihood (ML) algorithm [33] with MEGA 6.06 [34, 35]. Bootstrap analysis [36] with 100 replicates was performed to assess the support of the clusters

Phylogentic analysis of the single 16S rRNA gene copy affiliates *S. schinkii* strain Sp3 to the *Clostridia* class within the phylum *Firmicutes*. The RDP Classifier ([12] 2015-08-05) confirmed further the affiliation to *Thermoanaerobacteraceae* as published by [6] in 2011 (Table 1). The comparison of the 16S rRNA gene sequence with the latest available databases from GenBank (2015-08-05) using NCBI BLAST [13] under default settings identified the thermophilic SAOB *T. phaeum**(*NR_074723.1*)* as the closest characterized relative sharing 92.12 % identity (Fig. 2). *S. schinkii* is only distantly related to the characterized mesophilic SAOB *C. ultunense* ( 82.54 % identity), and *“T. acetatoxydans”* (84.1 % identity) and the thermophilic *P. lettingae* (79.64 %). Although *S. schinkii* has been physiologically affiliated to the group of acetogens, Fig. 2 illustrates a distant relationship to this group, as represented by e.g. the model acetogen *Moorella thermoacetica* (89.15 % identity).

## Genome sequencing information

### Genome project history

*Syntrophaceticus schinkii* strain Sp3 was sequenced and annotated by the SLU-Global Bioinformatics Centre at the Swedish University of Agricultural Sciences, Uppsala, Sweden. The genome project is deposited in the Genomes OnLine Database [14] with GOLD id Gi0035837 and the working draft genome is deposited in the European Nucleotide Archive database with accession number ERP005192. The SAOB was selected for sequencing on the basis of environmental relevance to issues in global carbon cycling, alternative energy production, and biochemical importance. Table2 contains the summary of project information.

**Table 1:** Classification and general features of *Syntrophaceticus schinkii* strain Sp3 according to the “minimum information about a Genome Sequence” (MIGS) specification [22]

MIGS ID	Property	Term	Evidence code^a^
	Classification	Domain *Bacteria*	TAS [23, 24]
		Phylum *Firmicutes*	TAS [25]
		Class *Clostridia*	TAS [26, 27]
		Order *Thermoanaerobacterales*	TAS [26] (p132), [28]
		Family *Thermoanaerobacteraceae*	TAS [26] (p132), [29]
		Genus *Syntrophaceticus*	TAS [6, 30]
		Species *Syntrophaceticus schinki*	TAS [6, 30]
		Strain *Sp3*	TAS [6]
	Gram stain	Variable	TAS [6]
	Cell shape	Variable ^b^	TAS [6]
	Motility	Non motile	TAS [6]
	Sporulation	Terminal endospores	TAS [6]
	Temperature range	Mesophilic	TAS [6]
	Optimum temperature	37–40 °C	TAS [6]
	Carbon source	Heterotroph	TAS [6]
	Energy source	Chemoheterotroph	TAS [6]
MIGS-6	Habitat	Anaerobic sludge	TAS [6]
MIGS-6.3	Salinity	Up to 0.6 M NH_4_Cl	TAS [6]
MIGS-22	Oxygen	Obligate anaerob	TAS [6]
MIGS-15	Biotic relationship	Syntrophy (beneficial)	TAS [6]
MIGS-14	Pathogenicity	Not reported	NAS
MIGS-4	Geographic location	Spain	NAS
MIGS-5	Sample collection time	1992	NAS
MIGS-4.1	Latitude	42.851329	NAS
MIGS-4.2	Longitude	−8.475933	NAS
MIGS-4.3	Depth	Not reported	NAS
MIGS-4.4	Altitude	Not reported	NAS

^a^Evidence codes—*TAS* Traceable Author Statement (i.e., a direct report exists in the literature), *NAS* Non-traceable Author Statement (i.e., not directly observed for the living, isolated sample, but based on a generally accepted property for the species, or anecdotal evidence). Evidence codes are from the Gene Ontology project [31]. ^b^Shape of cells varies between cocci and straight or slightly curved rods depend on NH_4_Cl concentration [6]

**Table 2:** Genome sequencing project information for the *Syntrophaceticus schinkii* Sp3 genome

MIGS ID	Property	Term
MIGS-31	Finishing quality	Draft
MIGD-28	Libraries used	Ion Torrent single end reads
MIGS-29	Sequencing platform	Ion Torrent PGM Systems
MIGS-31.2	Sequencing coverage	35×
MIGS-30	Assemblers	Newbler 2.8 and MIRA 4.0
MIGS-32	Gene calling method	PRODIGAL and AMIGene
	Locus Tag	SSCH
	Genbank ID	CDRZ00000000
	GenBank Data of release	March 21, 2014
	GOLD ID	Gi0035837
	BIOPROJECT	PRJNA224116
MIGS 13	Source Material Identifier	DSM 21860
	Project relevance	Biogas production

### Growth conditions and genomic DNA preparation

Since isolation by our research group, the strain has been kept in liquid cultures and a live culture and medium have been sent to DSMZ, (DSM21860). For DNA isolation batch cultures were grown in basal medium supplemented with 20 mM betaine as described by Westerholm et al. [6]. Cells were grown for 4 weeks at 37 °C without shaking and harvested at 5000 × g. DNA was isolated using the Blood & Tissue Kit from Qiagen (Hilden, Germany) according to the standard protocol recommended by the manufacturer.

### Genome sequencing and assembly

The genome of *Syntrophaceticus schinkii* was sequenced at the SciLifeLab Uppsala, Sweden using Ion torrent PM systems with the mean length of 206 bp, longest read length 392 bp and a total of final library reads of 2,985,963 for single end reads. All general aspects of sequencing performed can be found at Scilifelab website [15]. The FastQC software package [16] was used for reads quality assessment. After preassembly quality checking, the reads were assembled with MIRA 4.0 and Newbler 2.8 assemblers. Possible miss-assemblies were corrected manually by using Tablet, a graphical viewer for visualization of assemblies and read mappings [17]. A comparison of two assemblies obtained from both of the assemblers was used to fill the gaps between contigs. The multiple genome alignment tool Mauve was used for this purpose [18]. The working draft genome sequence of *S. schinkii* Sp3 contains 3,196,921 bp based on the analysis done with the tools summarized above.

### Genome annotation

Automated gene modeling was completed by MaGe [19] a bacterial genome annotation system. Genes were identified using Prodigal [20] and AMIGene [21] as part of MaGe genome annotation pipeline. The predicted CDSs were translated and used to search the NCBI non-redundant database, UniProt, TIGRFam, Pfam, PRIAM, KEGG, COG, and InterPro databases using BLASTP. Predicted coding sequences were subjected to manual analysis using MaGe web-based platform, which also provides functional information of proteins, and which was used to assess and correct genes predicted through the automated pipeline. The predicted functions were also further analyzed by the MaGe annotation system (Fig. 4).

## Genome properties

**Fig. 3:**
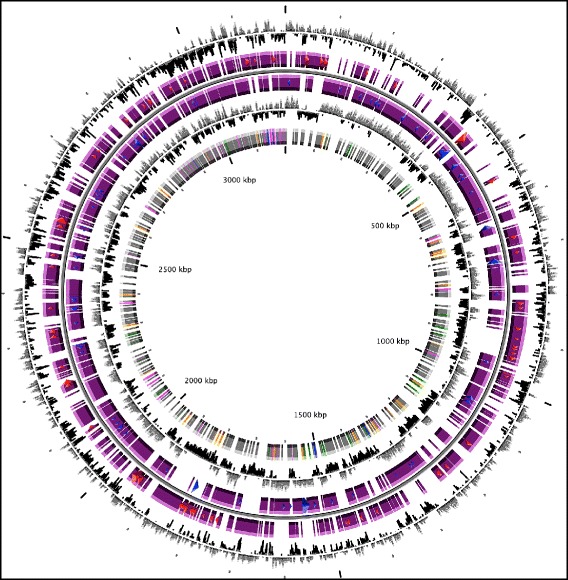
Circular map. Circular map of the *Syntrophaceticus schinkii Sp3 genome* (from the outside to the center): (1) GC percent deviation (GC window—mean GC) in a 1000-bp window. (2) Predicted CDSs transcribed in the clockwise direction. (3) Predicted CDSs transcribed in the counterclockwise direction. (4) GC skew (G + C/G-C) in a 1000-bp window. (5) rRNA (blue), tRNA (green), misc_RNA (orange), Transposable elements (pink) and pseudogenes (grey)

The working draft genome comprises 301 contigs in 215 scaffolds with a total size of 3,196,921 bp and a calculated GC content of 46.59 %. The genome shows a protein coding density of 75.21 % with an average intergenic length of 230.2 bp. The genome encodes further 50 tRNA genes and 5 rRNA genes, more precisely three 5S genes, one 16S and one 23S rRNA gene (Table 3, Fig. 3).

**Table 3:** Genomic statistics for the *Syntrophaceticus schinkii* strain Sp3 genome

Attribute	Value	% of total
Genome size (bp)	3,196,921	100.00
DNA Coding (bp)	2,399,289	75.05
DNA G + C content (bp)	1,489,445	46.59
Number of scaffolds	215	-
Total genes	3,441	100.00
Protein coding genes	3,281	95.35
RNA genes	55	1.59
Pseudo gene	90	2.61
Genes in internal clusters	2,086	60.62
Genes with function prediction	2,099	61.00
Genes assigned to COGs	2,583	75.07
Genes with Pfam domains	2,749	79.88
Genes with signal peptides	57	1.65
CRISPR repeats	8	.23

The genome of *S. schinkii* genome contains 3,441 predicted protein-encoding genes, of which 2,099 (61 %) have been assigned tentative functions. The remaining 1,346 ORFs are hypothetical / unknown proteins. 2,586 (app. 75 %) of all predicted protein-encoding genes could be allocated to the 22 functional COGs. This is in the same range as described for other acetogenic bacteria such as *Acetobacterium woodii* WB1 and *M. thermoacetica*ATCC39073, acetate oxidizing sulfate reducers such as *Desulfobacterium autotrophicum* HRM2 and *Desulfotomaculum kuznetsovii**,* and the SAOB *P.* lettingae TMO. Analysis of COGs revealed that ~28 % of all protein-encoding genes fall into four main categories: amino acid transport and metabolism (9.8 %), replication, recombination and repair (6.6 %), energy metabolism (5.9 %), and coenzyme transport and metabolism (4.9 %) (Table 4).

**Table 4:** Number of genes associated with the general COG functional categories

Code	Value	% age	Description
J	156	4.53	Translation, ribosomal structure and biogenesis
A	0	0.00	RNA processing and modification
K	211	6.12	Transcription
L	230	6.68	Replication, recombination and repair
B	1	0.03	Chromatin structure and dynamics
D	59	1.71	Cell cycle control, cell division, chromosome partitioning
Y	0	0.00	Nuclear structure
V	117	3.39	Defense mechanisms
T	136	3.95	Signal transduction mechanisms
M	169	4.90	Cell wall/membrane/envelope biogenesis
N	37	1.07	Cell motility
Z	1	0.02	Cytoskeleton
W	1	0.03	Extracellular structures
U	61	1.77	Intracellular trafficking, secretion, and vesicular transport
O	101	2.93	Posttranslational modification, protein turnover, chaperones
C	204	5.92	Energy production and conversion
G	138	4.00	Carbohydrate transport and metabolism
E	339	9.84	Amino acid transport and metabolism
F	70	2.03	Nucleotide transport and metabolism
H	172	4.99	Coenzyme transport and metabolism
I	52	1.51	Lipid transport and metabolism
P	206	5.98	Inorganic ion transport and metabolism
Q	54	1.57	Secondary metabolites biosynthesis, transport and catabolism
R	369	10.71	General function prediction only
S	219	6.36	Function unknown
	342	9.93	Not in COGs

## Insights from the genome sequence

**Fig. 4:**
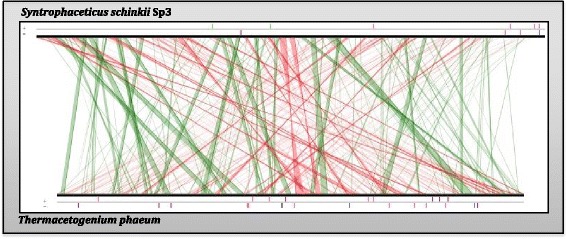
Synteny comparison. Synteny comparison of *S. schinkii* genome with the closely related genome of *T. phaeum*. Linear comparison of all predicted gene loci from *S. schinkii* with *T. phaeum* was perfomed using built-in tool in MaGe Platform with the synton size of > = 3 genes. The lines indicate syntons between two genomes. Red lines show inversions around the origin of replication. Vertical bars on the boarder line indicate different elements in genomes such as pink: transposases or insertion sequences: blue: rRNA and green: tRNA

Synteny-based analyses with all bacterial genomes present in the NCBI Reference Sequence database confirmed again that *T. phaeum* is the closest relative of *S. schinkii* having approximately 50 % of the total genome size in synteny (Fig. 4). A comparison of all inferred proteins of *S. schinkii* with all proteins collected in the NCBI RefSeq database revealed the highest number of orthologous (1788: 51.90 %) with *T. phaeum*. Both *S. schinkii* and *T. phaeum*, are known as syntrophic acetate oxidizing bacteria able to oxidize acetate in co-culture with a hydrogenotrophic methanogenic partner, but differ clearly in their substrate utilization patterns [2, 6] Moreover, in contrast to the thermophilic *T. phaeum*, *S. schinkii* possess mesophilic characteristics and cannot switch to a chemolithoautotrophic lifestyle.

The genome has been analyzed regarding general phenotypic features such as sporulation, oxygen tolerance, secreted and selenocystein-containing proteins and motility. The genome contains the master regulator Spo0A (SSCH_630004) needed for sporulation but lacks genes encoding the phosphorelays Spo0F and Spo0B as it has been observed in other clostridia. All the sporulation-specific sigma factors SigE (SSCH_460001), SigG (SSCH_1070017), and SigK (SSCH_700028) were predicted except for SigF. Two putative manganese containing catalases (SSCH_1760003, SSCH_2560004) and two putative rubrerythrin encoding genes (SSCH_590006, SSCH_180042) identified within the genome give reasons to believe, that this organism posses the ability to tolerate small amounts of oxygen. According to the observed immobility *S. schinkii* does not harbor any flagellum related genes including hook-associated proteins (FlgE, FlgK, FlgL), basal and hook proteins (FlgE), capping proteins (FliD), biosynthesis secretory proteins (FlhA, FlhB, FliF, FliH and FliI), flagella formation proteins, motor proteins (FliG and FliM) and the basal proteins (FlgC and FlgB).

Genes encoding key components of the selenocysteine-decoding (SelA, SelB, SelC, SelD) machinery are widely distributed in bacterial genomes. Also *S. schinkii* appears to have the ability to express selenocysteine proteins: The genome contains a single copy of the L-selenocysteinyl-tRNA^Sec^ transferase (selA: SSCH_110005/6), monoselenophosphate synthase (selD: SSCH_970007), the selenocysteinyl-tRNA specific elongation factor (selB: SSCH_110004) and potential selenocysteine-specific tRNA^Sec^ (selC: SSCH_tRNA31). We found two potential selenocysteine containing glycine/sarcosine/betaine reductase complexes encoded by the genome (SSCH_440002-8, SSCH_960012-15) consisting of selenoprotein subunit A, the substrate specific selenoprotein subunit B and acetyl phosphate forming subunit C. Since *S. schinkii* can only grow on betaine but not on glycine or sarcosine [6], this reductase complex might be specifically involved in betaine utilization. 57 CDSs were predicted to encode surface associated or secreted proteins identified by putative N-terminal signal peptides (signal peptide I and II).

## Conclusions

Acetate oxidation under anoxic conditions is thermodynamically unfavorable and requires the metabolic cooperation of a partner organism in order to make endergonic reactions more exergonic through the efficient removal of the products. *S. schinkii* oxidizes acetate to hydrogen and/or formate, which is directly used by a hydrogenotrophic methanogen. Since the methanogenic partner has been isolated and sequenced *S. schinkii* appears to have great potential to serve as a model organism for studying methane producing syntrophic relationships. The working draft genome sequence presented here will open the door for understanding the preferred habitats, the metabolism behind different life styles, and the mechanisms initiating syntrophy. This knowledge will help us to trigger SAOB towards an efficient and stable hydrogen/biogas production in engineered anaerobic digestion processes suffering high ammonia release.

## Additional file

Additional file 1:
**Table S1**. Associated MIGS record for *Syntrophaceticus schinkii* strain Sp3. (DOCX 111 kb)

## Abbreviations

SAOB: Syntrophic acetate-oxidizing bacteria
DSMZ: Deutsche Sammlung für Mikroorganismen und Zellkulturen
MIRA: Mimicking Intelligent Read Assembly
MaGe: Magnifying Genomes
BLASTP: Basic local alignment search tool for proteins
NCBI: National Center for Biotechnology Information
