# Supplementary material for: Working draft genome sequence of the mesophilic acetate oxidizing bacterium Syntrophaceticus schinkii strain Sp3
Source: Stand Genomic Sci. 2015 Nov 11;10:99. doi: 10.1186/s40793-015-0092-z (PMC4642661; doi:10.1186/s40793-015-0092-z)
Supplement: Additional file 1: — Table S1. Associated MIGS record for Syntrophaceticus schinkii strain Sp3. (DOCX 111 kb) [file 40793_2015_92_MOESM1_ESM.docx]

**Associated MIGS Record**

**Table S1.** Associated MIGS record.

| **MIGS-ID** | field name | description |
| --- | --- | --- |
| **MIGS-1** | Submit to INSDC/Trace archives |  |
| **1.1** | PID | PRJEB5769 |
| **1.2** | Trace Archive | CDRZ01000001-CDRZ01000301 |
| **MIGS-2** | MIGS CHECK LIST TYPE |  |
| **MIGS-3** | Project Name | Working draft genome sequence of the mesophilic SAOB (Acetate Oxidizing Bacterium) *Syntrophaceticus schinkii* strain Sp3 |
| **MIGS-4** | Geographic Location | Spain |
| **4.1** | Latitude | 42.851329 |
| **4.2** | Longitude | -8.475933 |
| **4.3** | Depth | Not reported |
| **4.4** | Altitude | Not reported |
| **MIGS-5** | Time of Sample collection | Not reported |
| **MIGS-6** | Habitat (EnvO) | Anaerobic sludge |
| **6.1** | temperature | Mesophilic |
| **6.2** | pH | Not reported |
| **6.3** | salinity | Not reported |
| **6.4** | chlorophyll | Not reported |
| **6.5** | conductivity | Not reported |
|  |  |  |
| **6.6** | light intensity | Not reported |
| **6.7** | dissolved organic carbon (DOC) | Not reported |
| **6.8** | current | Not reported |
| **6.9** | atmospheric data | Not reported |
| **6.10** | density | Not reported |
| **6.11** | alkalinity | Not reported |
| **6.12** | dissolved oxygen | Not reported |
| **6.13** | particulate organic carbon (POC) | Not reported |
| **6.14** | phosphate | Not reported |
| **6.15** | nitrate | Not reported |
| **6.16** | sulfates | Not reported |
| **6.17** | sulfides | Not reported |
| **6.18** | primary production | Not reported |
| **MIGS-7** | Subspecific genetic lineage |  |
| **MIGS-9** | Number of replicons | 01 |
| **MIGS-10** | Extrachromosomal elements | None |
| **MIGS-11** | Estimated Size | 3.1 Mbp |
| **MIGS-12** | Reference for biomaterial or Genome report |  |
| **MIGS-13** | Source material identifiers |  |
| **MIGS-14** | Known Pathogenicity | None |
|  |  |  |
| **MIGS-15** | Biotic Relationship | Syntrophy |
| **MIGS-16** | Specific Host | None |
| **MIGS-17** | Host specificity or range (taxid) |  |
| **MIGS-18** | Health status of Host |  |
| **MIGS-19** | Trophic Level |  |
| **MIGS-22** | Relationship to Oxygen | Obligate anaerobe |
| **MIGS-23** | Isolation and Growth conditions |  |
| **MIGS-27** | Nucleic acid preparation |  |
| **MIGS-28** | Library construction |  |
| **28.1** | Library size | 4,866,824 (98%) |
| **28.2** | Number of reads | 2,985,963 (61 %) |
| **28.3** | vector |  |
| **MIGS-29** | Sequencing method | Ion Torrent |
| **MIGS-30** | Assembly | SSCH |
| **30.1** | Assembly method | *De novo* |
| **30.2** | estimated error rate |  |
| **30.3** | method of calculation |  |
| **MIGS-31** | Finishing strategy |  |
| **31.1** | Status | Working draft |
| **31.2** | coverage | 62 |
| **31.3** | contigs | 301 |
| **MIGS-32** | Relevant SOPs |  |
| **MIGS-33** | Relevant e-resources |  |
